# Amygdala Dopamine Receptors Are Required for the Destabilization of a Reconsolidating Appetitive Memory^1^,^2^

**DOI:** 10.1523/ENEURO.0024-14.2015

**Published:** 2015-03-06

**Authors:** Emiliano Merlo, Patrizia Ratano, Elena C. Ilioi, Miranda A.L.S. Robbins, Barry J. Everitt, Amy L. Milton

**Affiliations:** Department of Psychology, University of Cambridge, Cambridge CB2 3EB, United Kingdom

**Keywords:** amygdala, dopamine, reconsolidation

## Abstract

Disrupting maladaptive memories may provide a novel form of treatment for neuropsychiatric disorders, but little is known about the neurochemical mechanisms underlying the induction of lability, or destabilization, of a retrieved consolidated memory. Destabilization has been theoretically linked to the violation of expectations during memory retrieval, which, in turn, has been suggested to correlate with prediction error (PE). It is well-established that PE correlates with dopaminergic signaling in limbic forebrain structures that are critical for emotional learning. The basolateral amygdala is a key neural substrate for the reconsolidation of pavlovian reward-related memories, but the involvement of dopaminergic mechanisms in inducing lability of amygdala-dependent memories has not been investigated. Therefore, we tested the hypothesis that dopaminergic signaling within the basolateral amygdala is required for the destabilization of appetitive pavlovian memories by investigating the effects dopaminergic and protein synthesis manipulations on appetitive memory reconsolidation in rats. Intra-amygdala administration of either the D1-selective dopamine receptor antagonist SCH23390 or the D2-selective dopamine receptor antagonist raclopride prevented memory destabilization at retrieval, thereby protecting the memory from the effects of an amnestic agent, the protein synthesis inhibitor anisomycin. These data show that dopaminergic transmission within the basolateral amygdala is required for memory labilization during appetitive memory reconsolidation.

## Abstract (precis)

Memories are not fixed in the brain, but undergo experience-dependent updating and modification through reconsolidation. This occurs when a memory is converted to a labile state, usually involving surprise (formally, prediction error), which is in turn linked to release of dopamine.

## Significance Statement

Memories are not fixed in the brain, but undergo experience-dependent updating and modification through reconsolidation. This occurs when a memory is converted to a labile state, usually involving surprise (formally, prediction error), which is in turn linked to release of dopamine. We hypothesized that neurotransmission via dopamine receptors in the amygdala, a region critical for emotional memory processing, is required for memory destabilization. The results show that blocking dopamine receptors in the amygdala protected reward-associated memories from an amnestic treatment. Therefore, dopamine is required for the induction of pavlovian memory lability, supporting a link between destabilization and prediction error. Thus, dopaminergic signaling allows memories to be dynamic and flexible, providing a novel target for the modification of maladaptive memories.

## Introduction

Reconsolidation is the process by which memories become destabilized at retrieval and subsequently restabilize in order to persist in the brain. This process has received much interest for its potential as a novel treatment target for neuropsychiatric disorders such as drug addiction (Milton and Everitt, 2012; Tronson and Taylor, 2013) and post-traumatic stress disorder (PTSD; Dębiec and LeDoux, 2006). Both addiction and PTSD can be conceptualized as disorders of maladaptive emotional memory, depending critically upon areas of the limbic forebrain such as the basolateral amygdala (BLA; Everitt et al., 2000; Cardinal et al., 2002), which is required for the storage of memories associating a conditioned stimulus (CS) with its emotional and motivational affective value (Weiskrantz, 1956) imbued by association with an unconditioned stimulus (US). However, although the mechanisms underlying the restabilization of CS−US memories are increasingly understood (Nader et al., 2000; Bozon et al., 2003; Lee et al., 2005; von Hertzen and Giese, 2005), less is known about the mechanisms responsible for triggering memory lability, or memory destabilization (Finnie and Nader, 2012). The mechanisms underlying memory retrieval and destabilization are not necessarily identical (Forcato et al., 2009; Milton et al., 2013). Even if retrieved, without destabilization, a memory will remain insensitive to any attempt to disrupt it by applying treatments that would prevent its restabilization, such as protein synthesis inhibitors. Therefore, understanding the specific mechanisms that underlie memory destabilization is important from both basic science and translational perspectives.

The induction of memory lability has been theoretically linked to the construct of prediction error (PE) in invertebrates (Pedreira et al., 2004; Eisenhardt and Menzel, 2007), rats (Lee, 2009), and humans (Forcato et al., 2009; Sevenster et al., 2013). PE correlates with activity in midbrain dopaminergic neurons (Schultz et al., 1997). Although dopamine has been most consistently implicated in the coding of a reward PE (Schultz et al., 1993), with omissions of reward being associated with reductions in dopaminergic cell firing (Schultz et al., 1997), there is evidence to suggest that a population of dopamine neurons in the ventral tegmental area (VTA) increase their firing in response to the presentation of aversive stimuli (Brischoux et al., 2009) and negative prediction errors, especially in the context of pavlovian overexpectation (Takahashi et al., 2009).

The BLA receives its dopaminergic innervation from the VTA (Asan, 1997; Brinley-Reed and McDonald, 1999), and the major subtypes of dopamine receptor (the D_1_-like and D_2_-like) are expressed within the BLA (Maltais et al., 2000). Furthermore, electrophysiological responses of BLA neurons to VTA dopamine release are consistent with a role in encoding prediction error, conforming to “surprise” in Pearce-Kaye-Hall models of learning (Esber et al., 2012). Inactivation of the VTA prevents the destabilization of the memories underlying pavlovian-conditioned approach (Reichelt et al., 2013), but it is unlikely that the VTA is itself the site of memory storage. Previous work has demonstrated the necessity of the amygdala for the reconsolidation of pavlovian reward-associated memories (Lee et al., 2006; Milton et al., 2008; Sanchez et al., 2010; Théberge et al., 2010; Barak et al., 2013; Wells et al., 2013; Arguello et al., 2014; Olshavsky et al., 2014), with different amygdala subnuclei likely supporting different pavlovian reward-associated processes (Milton and Everitt, 2010). Reward-associated pavlovian CSs presented in the absence of reward (as occurs during memory reactivation) increase markers of dopamine release in the amygdala in rats (Harmer and Phillips, 1999) and human subjects (Fotros et al., 2013). The BLA itself is also responsive to both positive and negative prediction errors (Roesch et al., 2010; Tye et al., 2010), leading us to speculate that the destabilization of the memory underlying conditioned reinforcement, induced by the omission of reward during the early termination of a memory reactivation session relative to expectations generated by previous training, may be dependent upon dopaminergic signaling originating in the VTA. To date, there has been no direct test of the hypothesis that dopaminergic signaling within the BLA is required for memory destabilization. Although it has previously been shown that antagonism at D_1_ dopamine receptors can disrupt the reconsolidation of a passive avoidance memory in chicks (Sherry et al., 2005) and that systemic antagonism at D_1_ or D_3_ dopamine receptors impaired the reconsolidation of memories underlying cocaine-associated behavior in mice (Yan et al., 2013; Yan et al., 2014), it is not clear where these manipulations are acting in the brain to exert these effects on the reconsolidating memory, nor the precise component process—destabilization or restabilization—that is affected by the dopaminergic transmission event. Furthermore, blocking signaling at D_1_/D_5_ dopamine receptors specifically in the hippocampus prevented the destabilization of object recognition memory (Rossato et al., 2014), illustrating the complex effects of dopaminergic manipulations on the engram, including the difference between systemic and central dopamine receptor antagonism.

Therefore, based on the theoretical links between unsigned prediction error, dopaminergic signaling, and memory destabilization, the hypothesis tested in these experiments was that the destabilization of the memory that underlies the capacity of a pavlovian CS to act as a conditioned reinforcer, known to depend upon the BLA (Burns et al., 1999), would be prevented by antagonism at BLA dopamine receptors. This hypothesis was tested by measuring in separate experiments the effects of intra-BLA infusions of the D_1_ receptor (D_1_R) antagonist SCH23390, the D_2_R antagonist raclopride, and the non-subtype selective dopamine receptor antagonist α-flupenthixol on the destabilization of an appetitive CS−sucrose memory, assessed through a procedure that selectively measures the conditioned reinforcing properties of pavlovian CSs. To test whether memory destabilization had occurred, the capacity of the protein synthesis inhibitor anisomycin, given immediately after memory reactivation, to induce subsequent amnesia was assessed, as described previously (Ben Mamou et al., 2006; Milton et al., 2013).

## Materials and Methods

### Subjects

Subjects were 77 experimentally naïve male Lister-Hooded rats (Charles River) housed in pairs in a vivarium on a reversed light−dark cycle (lights on at 1900 hours). Subjects weighed at least 290 g prior to surgery. Subjects were food restricted, though not deprived, being maintained at at least 90% of free-feeding weight and fed after training or testing each day. Access to water was *ad libitum* except for when inside the conditioning chambers. All procedures were conducted in accordance with the UK Animals (Scientific Procedures) Act 1986.

### Surgery

**Figure 1:**
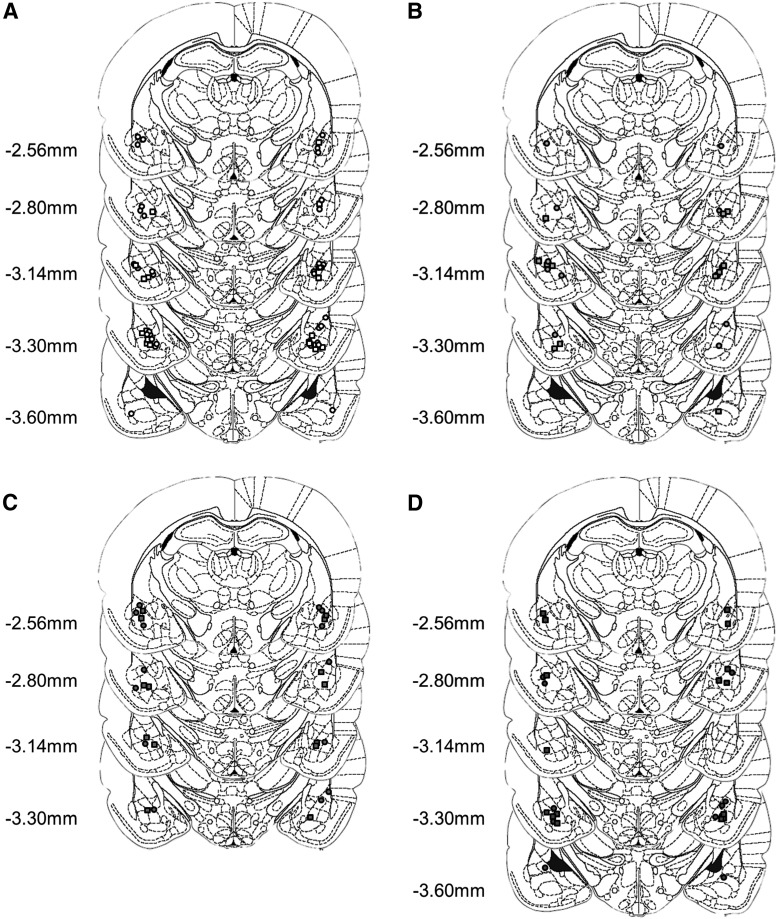
Injector tips were located within the BLA for animals receiving vehicle (***A***), SCH23390 (***B***), raclopride (***C***), and α-flupenthixol (***D***) prior to memory reactivation. Circles represent animals that received vehicle following memory reactivation, and squares the placements for the animals that received anisomycin following reactivation. Distances are given from bregma. This figure was modified, with permission, from Paxinos & Watson (2004).

Rats were implanted with bilateral guide cannulae (16 mm, 24 gauge; Coopers Needle Works) located just dorsal to the BLA (Fig. 1) (Milton et al., 2008). The coordinates for cannula implantation were AP −2.6 mm and ML ±4.5 mm (relative to bregma) and DV −5.6 mm (relative to dura). A recovery period of at least 7 d was given before behavioral training and testing began.

### Intracerebral drug administration

Infusions were carried out using a syringe pump (Harvard Apparatus) and 5 μl Hamilton syringes, connected to injectors (28 gauge, projecting 2 mm beyond the guide cannulae; Plastics One) by polyethylene tubing. The rats received two infusions; one immediately prior to the memory reactivation session, and one immediately afterwards. All infusions were begun 30 s after the insertion of the injectors and performed over 2 min at a rate of 0.25 μl min^−1^ (total volume of 0.5 μl side^−1^). One minute of waiting time was imposed from the end of the infusion to the removal of the injectors to allow diffusion of the solution away from the infusion site. Although we did not test whether the infusions were restricted to the BLA, it is the basolateral nucleus, rather than the adjacent central nucleus, that has been implicated in supporting the conditioned reinforcing properties of pavlovian CSs (Burns et al., 1999).

### Drugs

All rats received either the protein synthesis inhibitor anisomycin (ANI) or its vehicle (VEH) as their second (post-reactivation) infusion. Anisomycin (125 μg μl^−1^; Sigma-Aldrich) was dissolved in equimolar HCl and then pH-balanced to pH 7.4 with NaOH. This dose of anisomycin has previously been shown to disrupt memory reconsolidation (Ben Mamou et al., 2006; Milton et al., 2013).

Prior to memory reactivation, rats received an infusion of either drugs targeting the dopaminergic signaling system or the PBS vehicle. α-(cis)-flupenthixol (FLU; Sigma-Aldrich) was dissolved in PBS at a concentration of 20 μg μl^−1^, which has been shown to reduce cue-maintained cocaine-seeking when infused into the BLA (Di Ciano and Everitt, 2004) without nonspecific locomotor effects. The D_1_-selective dopamine receptor antagonist SCH23390 (SCH; Tocris Bioscience) was dissolved in PBS at a concentration of 4 μg μl^−1^ and the D_2_-selective receptor antagonist raclopride (RAC; Sigma-Aldrich) was dissolved in PBS at a concentration of 10 μg μl^−1^. These doses of SCH and RAC have been shown to be effective in blocking the consolidation of inhibitory avoidance memory (LaLumiere et al., 2004) and cue-induced reinstatement of drug-seeking (Alleweireldt et al., 2006; Berglind et al., 2006).

### Behavioral procedures

All behavioral procedures were conducted during the animals’ dark cycle. Rats were trained in conditioning chambers (Med Associates) to make a nosepoke response into a central magazine for presentation of a 0.1 ml of a 10% sucrose reinforcer (Tate & Lyle), which was associated with a 10 s light CS (presented on the same side assigned to the inactive lever during testing, counterbalanced across rats) on a fixed ratio (FR) 1 schedule. Rats were trained over nine sessions, with a maximum of 30 CS−sucrose pairings per session.

The day after the completion of training, rats received intra-BLA infusions of either the dopamine receptor antagonists (FLU, SCH, or RAC) or VEH, and immediately (within 1 − 2 min) afterwards began a 15 min memory reactivation session. During this session, nosepokes led to the presentation of the light CS and movement of the dipper on an FR1 schedule, but no sucrose was delivered. The rats were limited to a maximum of 30 CS presentations during this session, but the 15 min session normally terminated before this limit was reached. Immediately after the end of the memory reactivation session, the rats received a second intra-BLA infusion of either ANI or its VEH.

Acquisition of a new instrumental response for conditioned reinforcement (ANR) testing began 24 h after the memory reactivation session. The rats were returned to the same conditioning chambers, but in this phase they were presented with two novel levers (left and right of the central magazine). Depression of the active lever led to an abbreviated (1 s) presentation of the light CS on a variable ratio (VR1-3) schedule, while depression of the inactive lever had no programmed consequence and acted as a control for general activity. The light CS was always presented on the side opposite to the active lever to avoid pavlovian-conditioned approach contributing to lever pressing. No sucrose was available during these sessions. Rats were returned to the chambers for seven 30 min sessions, conducted 1, 2, 5, and 8 d following memory reactivation, and then weekly following day 8 (on days 15, 22, and 29). Lever presses and nosepokes were recorded by computer.

### Histology

Following the end of testing, rats were killed with an overdose of anesthetic (Dolethal; Vétoquinol) and transcardially perfused with 0.01 M PBS through the ascending aorta, followed by 4% paraformaldehyde. The brains were removed and stored in 4% paraformaldehyde for at least 24 h, before being transferred to a 20% sucrose solution for cryoprotection prior to sectioning. The brains were sectioned at 60 μm, mounted on microscope slides, and stained with Cresyl Violet. Cannulae placements (Fig. 1) were verified by light microscopy (Leica) and any subjects for which the injector tips were located outside the basolateral amygdala were excluded from all analyses.

### Sample size, statistical power, and randomization

*A priori* sample size calculations were not conducted but the number of subjects per group was chosen by reference to previous research. Data was collected over an extended period of time, with eight animals being run within a single squad. Since there was no effect of squad on the number of nosepokes at training (TR) or reactivation (React) (Squad_TR_: *F*_(17,59)_ = 1.15, *p* = 0.33, Squad_React_: *F*_(17,59)_ = 1.54, *p* = 0.11) or on the number of lever presses during the ANR test (Squad: *F*_(17,59)_ = 1.08, *p* = 0.39), data from different squads were pooled for analysis. Subjects were pseudo-randomly assigned to experimental groups, such that an individual squad received as a first infusion only a subset of the drugs under investigation (i.e. animals within an individual squad received only FLU or VEH, SCH or VEH, or RAC or VEH). All squads contained animals receiving VEH or ANI for the second infusion. Drug assignments were also made such that training performance was matched across experimental groups.

### Data collection and statistical analysis

Data were recorded automatically by the Conditioned Reinforcement program (Cardinal, 2005) running within the Whisker Control server (Cardinal, 2000). As the data were collected by computer, blinding to experimental group was not required.

Training and testing data were analyzed using repeated-measures ANOVA, and reactivation data were analyzed using a one-way ANOVA. The normality assumption of ANOVA was checked with the Shapiro-Wilk test, and if this indicated that the data were not normally distributed then they were transformed. This was the case for the nosepoke data from training, which was transformed using the Box-Cox method with λ = −0.5; i.e. using the equation *y* = 1/(√*x*), where *x* is the original data and *y* the transformed value. Following this transformation, the data satisfied the assumption of normality (all *W* > 0.97, all *p* > 0.058). The lever pressing and nosepoke data from the ANR phase of the experiment were also not normally distributed, so were transformed using the Box-Cox method with λ = 0.5; i.e. square-root transformed. Following this transformation, the majority of the lever press data satisfied the assumption of normality (*p* > 0.05).

If Mauchly’s test indicated that the assumption of sphericity had been violated, then the Greenhouse-Geisser correction was applied where ε < 0.75, and the Huynh-Feldt correction applied where ε > 0.75, as recommended by Cardinal and Aitken (2006). The α level was 0.05 for all analyses, and *p* values are two-tailed. Where appropriate, subsequent ANOVAs and Šidák-corrected pairwise comparisons were conducted to investigate specific *a priori* hypotheses. For ease of interpretation, we have represented the factor names by the name of the drug infused when reporting the statistics (e.g., Drug2 is shown as “ANI”) in Table 1.

**Table 1:** Statistics

Analysis	Effect	Outcome	Data structure	Type of test	Power (α = 0.05)
NP at TR	squad	*F*_(17,59)_ = 1.15	a	2	0.681
	session	*F*_(6.1,421)_ = 13.0	a	1	1
	session × drug1	*F*_(18,421)_ = 1.59	a	1	0.935
	session × drug2	*F*_(6.1,421)_ = 1.25	a	1	0.499
	session × drug1 × drug2	*F* < 1	a	1	0.617
	drug1	*F* < 1	a	1	0.089
	drug2	*F* < 1	a	1	0.052
	drug1 × drug2	*F* < 1	a	1	0.096
CS at TR	session	*F*_(8,552)_ = 1.20	b	1	0.559
	drug1	*F* < 1	b	1	0.234
	drug2	*F*_(1,69)_ = 2.52	b	1	0.346
	drug1 × drug2	*F* < 1	b	1	0.234
	session × drug1	*F*_(24,552)_ = 1.56	b	1	0.971
	session x drug1 x drug2	*F*_(24,552)_ = 1.56	b	1	0.971
NP at React	squad	*F*_(17,59)_ = 1.54	b	2	0.836
	drug1	*F* < 1	b	2	0.159
CS at React	drug1	*F*_(3,76)_ = 1.45	b	2	0.37
NP at React	drug2	*F* < 1	b	3	0.077
	drug1 × drug2	*F* < 1	b	3	0.146
CS at React	drug2	*F* < 1	b	3	0.086
	drug1 × drug2	*F* < 1	b	3	0.156
LP at ANR	squad	*F*_(17,59)_ = 1.08	a	2	0.644
	drug1 × drug2	*F*_(3,69)_ = 3.40	a	1	0.743
	lever × drug1 × drug2	*F*_(38.4,69.0)_ = 3.11	a	1	0.701
	drug1	*F*_(3,69)_ = 1.24	a	1	0.319
	lever × drug1	*F*_(14.4,69.0)_ = 1.16	a	1	0.3
	drug2	*F*_(1,69)_ = 2.67	a	1	0.364
	lever × drug2	*F* < 1	a	1	0.052
NP at ANR	drug1	*F* < 1	a	1	0.116
	drug2	*F*_(1,69)_ = 1.44	a	1	0.212
	drug1 × drug2	*F*_(3,69)_ = 1.72	a	1	0.498
	session × drug1	*F*_(16.7,386)_ = 1.71	a	1	0.941
LP/VEH	ANI	*F*_(1,33)_ = 5.33	a	1	0.61
	lever × session in VEH/VEH	*F*_(3.6,89.6)_ = 6.27	a	1	0.978
	lever × session in VEH/ANI	*F*_(2.4,19.4)_ = 1.8	a	1	0.36
NP/VEH	ANI	*F*_(1,33)_ = 2.05	a	1	0.285
	session × ANI	*F* < 1	a	1	0.265
LP/SCH	SCH × ANI	*F*_(1,45)_ = 6.75	a	1	0.720
	ANI	*F* < 1	a	1	0.131
	lever × ANI	*F* < 1	a	1	0.078
	lever × session × ANI	*F*_(2.99,35.9)_ = 1.60	a	1	0.384
	lever	*F*_(1,12)_ = 36.7	a	1	1
	lever × session	*F*_(2.99,35.9)_ = 3.88	a	1	0.78
NP/SCH	ANI	*F*_(1,12)_ = 1.55	a	1	0.209
	session × ANI	*F* < 1	a	1	0.124
LP/RAC	RAC × ANI	*F*_(1,46)_ = 7.26	a	1	0.751
	lever	*F*_(1,13)_ = 23.1	a	1	0.993
	lever × ANI	*F*_(1,13)_ = 6.04	a	1	0.623
	ANI	*F* < 1	a	1	0.081
NP/RAC	ANI	*F*_(1,13)_ = 2.64	a	1	0.324
	session × ANI	*F* < 1	a	1	0.319
LP/FLU	FLU × ANI	*F*_(1,44)_ = 2.26	a	1	0.312
	ANI	*F*_(1,11)_ = 7.48	a	1	0.702
NP/FLU	ANI	*F*_(1,11)_ = 1.36	a	1	0.187
	session × ANI	*F* < 1	a	1	0.231
LP/Drug1	drug1	*F* < 1	a	1	0.249
	lever × drug1	*F*_(3,43)_ = 1.43	a	1	0.352
	session × drug1	*F*_(15.6,224)_ = 1.11	a	1	0.72
	lever × session × drug1	*F* < 1	a	1	0.5
LP/ANI	drug1	*F*_(3,26)_ = 3.01	a	1	0.639
	lever × drug1	*F*_(3,26)_ = 2.53	a	1	0.557
VEH/ANI	lever	*F*_(1,8)_ = 2.13	a	1	0.251
FLU/ANI	lever	*F*_(1,7)_ = 3.06	a	1	0.327
SCH/ANI	lever	*F*_(1,5)_ = 29.6	a	1	0.989
RAC/ANI	lever	*F*_(1,6)_ = 39.6	a	1	0.999

a, Normal distribution after transformation; b, normal distribution; 1, repeated-measures ANOVA; 2, one-way ANOVA; 3, two-way ANOVA. NP, nosepoke; TR, training session; React, memory reactivation session; LP, lever pressing; ANR, acquisition of a new response.

## Results

### All experimental groups readily acquired the CS−sucrose association during training

Briefly, animals were trained over nine sessions in conditioning chambers to make an instrumental nosepoke response into a magazine to receive presentations of 10% liquid sucrose and a pavlovian light CS, with a maximum of 30 CS−sucrose presentations per day. All animals readily acquired this task, with no differences in the performance of the animals prospectively assigned to different experimental groups (Table 2) during training. Due to a non-normal distribution, instrumental training data were transformed prior to analysis (see Materials and Methods), and analysis of these transformed data revealed that instrumental responding increased across the training sessions (Session: *F*_(6.1,421)_ = 13.0, *p* < 0.001, η^2^ = 0.16), though with no differences in instrumental responding between the prospective experimental groups assigned to receive dopamine receptor antagonists or vehicle prior to reactivation (Drug1) and anisomycin or vehicle after reactivation (Drug2) (Session × Drug1: *F*_(18,421)_ = 1.59, *p* = 0.058; Session × Drug2: *F*_(6.1,421)_ = 1.25, *p* = 0.28; Session × Drug1 × Drug2: *F* < 1; Drug1: *F* < 1; Drug2: *F* < 1; Drug1 × Drug2: *F* < 1). Furthermore, as the test data for each experiment were analyzed separately, follow-up ANOVAs were conducted and revealed no differences in training performance between groups assigned to receive ANI or its VEH following memory reactivation (e.g., no difference in the behavior of animals assigned to the VEH/VEH condition vs the VEH/ANI condition) for any of the experimental groups receiving a dopamine receptor antagonist (SCH, RAC, or FLU) or VEH prior to reactivation (all *p*’s > 0.37). Therefore, all experimental groups were equivalent on the basis of their training performance.

**Table 2:** **Training performance was equivalent across experimental groups** Data are presented as mean ± SEM and where appropriate are given to 3 significant figures

Session	1	2	3	4	5	6	7	8	9
CSs									
VEH/VEH	30 ± 0	30 ± 0	30 ± 0	30 ± 0	30 ± 0	30 ± 0	30 ± 0	30 ± 0	30 ± 0
VEH/ANI	30 ± 0	30 ± 0	30 ± 0	30 ± 0	30 ± 0	29.7 ± 0.33	30 ± 0	30 ± 0	30 ± 0
SCH/VEH	30 ± 0	30 ± 0	30 ± 0	30 ± 0	30 ± 0	30 ± 0	30 ± 0	30 ± 0	30 ± 0
SCH/ANI	30 ± 0	30 ± 0	30 ± 0	30 ± 0	30 ± 0	30 ± 0	30 ± 0	30 ± 0	30 ± 0
RAC/VEH	30 ± 0	30 ± 0	30 ± 0	30 ± 0	30 ± 0	30 ± 0	30 ± 0	30 ± 0	30 ± 0
RAC/ANI	30 ± 0	30 ± 0	30 ± 0	30 ± 0	30 ± 0	30 ± 0	30 ± 0	30 ± 0	29.4 ± 0.57
FLU/VEH	30 ± 0	30 ± 0	30 ± 0	30 ± 0	30 ± 0	30 ± 0	30 ± 0	30 ± 0	30 ± 0
FLU/ANI	30 ± 0	30 ± 0	30 ± 0	30 ± 0	30 ± 0	30 ± 0	30 ± 0	30 ± 0	30 ± 0
Nosepokes									
VEH/VEH	80.2 ± 2.37	70.5 ± 2.67	64.8 ± 2.33	60.4 ± 2.47	62.8 ± 2.95	66.5 ± 3.06	72.8 ± 5.33	72.2 ± 4.28	82.3 ± 5.66
VEH/ANI	86.6 ± 7.01	65.3 ± 3.54	62.2 ± 4.16	62.3 ± 3.72	61.0 ± 2.86	63.9 ± 6.38	72.6 ± 4.36	70.7 ± 6.26	79.9 ± 6.75
SCH/VEH	85.5 ± 7.53	80.1 ± 10.8	72.0 ± 8.79	69.6 ± 11.4	77.0 ± 16.0	81.5 ± 16.5	77.8 ± 11.7	68.9 ± 12.3	70.1 ± 9.76
SCH/ANI	88.5 ± 7.48	80.7 ± 5.44	65.7 ± 4.55	70.8 ± 2.09	72.8 ± 6.52	73.0 ± 6.96	69.0 ± 6.52	57.5 ± 4.16	85.3 ± 8.09
RAC/VEH	82.5 ± 4.15	73.3 ± 4.85	71.8 ± 7.56	74.5 ± 5.61	60.5 ± 6.04	69.6 ± 3.67	65.1 ± 4.67	66.0 ± 6.39	77.8 ± 7.41
RAC/ANI	79.3 ± 3.57	73.6 ± 5.55	67.0 ± 2.27	63.9 ± 1.71	58.7 ± 3.94	57.3 ± 3.41	59.9 ± 7.48	73.1 ± 12.8	81.1 ± 16.6
FLU/VEH	79.6 ± 8.59	73.4 ± 13.53	68.2 ± 7.86	64.0 ± 7.56	58.0 ± 8.13	65.6 ± 5.45	63.4 ± 9.69	82.0 ± 7.50	67.6 ± 8.33
FLU/ANI	84.6 ± 5.73	64.8 ± 4.05	73.3 ± 6.52	67.4 ± 5.93	63.4 ± 6.67	64.0 ± 3.49	69.0 ± 5.15	66.5 ± 4.98	84.3 ± 7.44

All groups also received equal numbers of CS presentations during training, with all except two animals reaching the limit of 30 CS−sucrose presentations in every training session (Table 2). All animals acquired the task rapidly, receiving the maximum number of CS presentations from the first training session onwards (Session: *F*_(8,552)_ = 1.20, *p* = 0.297). There were no differences in the number of CS presentations between the prospective experimental groups (Drug1: *F* < 1; Drug2: *F*_(1,69)_ = 2.52, *p* = 0.12; Drug1 × Drug2: *F* < 1). Due to two rats receiving fewer than 30 CS presentations in one session of training, there were significant interactions of Session × Drug1 (*F*_(24,5520)_ = 1.56, *p* = 0.045, η^2^ = 0.06) and Session × Drug1 × Drug2 (*F*_(24,552)_ = 1.56, *p* = 0.045, η^2^ = 0.06), but it is unlikely that these very small effects are biologically meaningful.

### Behavior during the memory reactivation session was not acutely affected by the administration of dopamine receptor antagonists

**Figure 2:**
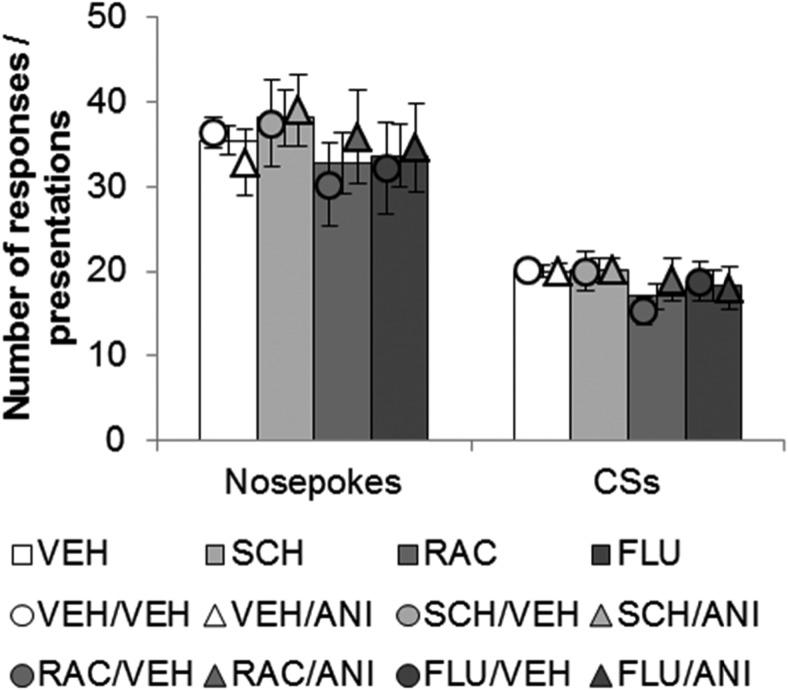
Dopamine receptor antagonism had no acute effects on behavior during the memory reactivation session. There were no differences between experimental groups in the number of nosepoke responses made or the number of CSs obtained during the memory reactivation session. Thus, dopamine receptor antagonism with SCH23390 (SCH), raclopride (RAC), or α-flupenthixol (FLU) did not acutely affect activity or memory retrieval relative to vehicle (VEH). Data are presented as means ± SEM. The bars represent data for all animals receiving the same infusion prior to reactivation; the circles and triangles represent data for the prospective experimental groups, based on the second infusion of anisomycin or vehicle following the reactivation session. Group sizes: VEH/VEH = 26; VEH/ANI = 9; SCH/VEH = 8; SCH/ANI = 6; RAC/VEH = 8; RAC/ANI = 7; FLU/VEH = 5; FLU/ANI = 8 rats per group.

Dopamine receptor antagonists applied to the BLA immediately before the reactivation session affected neither the retrieval of the CS−sucrose memory, nor had any generalized locomotor effects (Fig. 2). None of the dopamine receptor antagonists administered prior to memory reactivation affected the number of nosepokes made (Drug1: *F* < 1) or the number of CSs presented (Drug1: *F*_(3,76)_ = 1.45, *p* = 0.23) during the reactivation session itself. Furthermore, there were no differences in the reactivation experience of those animals receiving VEH or ANI immediately after the memory reactivation session in terms of the number of nosepokes made (Drug2: *F* < 1; Drug1 × Drug2: *F* < 1) or the number of CSs presented (Drug2: *F* < 1; Drug1 × Drug2: *F* < 1), suggesting that these prospective experimental groups were well-matched for behavior.

Further analysis of the data indicated that for all experimental groups receiving different pre-reactivation infusions (VEH, SCH, RAC, or FLU), analyzed separately, there were no differences in the performance at reactivation of animals receiving ANI or VEH following reactivation (e.g., no differences in behavior of animals in the VEH/VEH condition vs the VEH/ANI condition) when assessed through the number of nosepokes made during memory reactivation (all *p*’s > 0.37) or the number of CS presentations (all *p*’s > 0.24).

### Dopamine receptor antagonism prior to memory reactivation blocked the amnestic effect of the protein synthesis inhibitor anisomycin administered after reactivation

To evaluate the integrity of the pavlovian CS−sucrose association after the reactivation session, the conditioned reinforcing property of the light CS was assessed through its ability to support the ANR (Mackintosh, 1974). The absence of discriminated responding between active and inactive levers across sessions would imply that the CS−sucrose association had been disrupted by ANI infused into the BLA at reactivation. An amnestic effect of ANI would only be seen if the CS−sucrose memory had been destabilized during the reactivation session.

**Figure 3:**
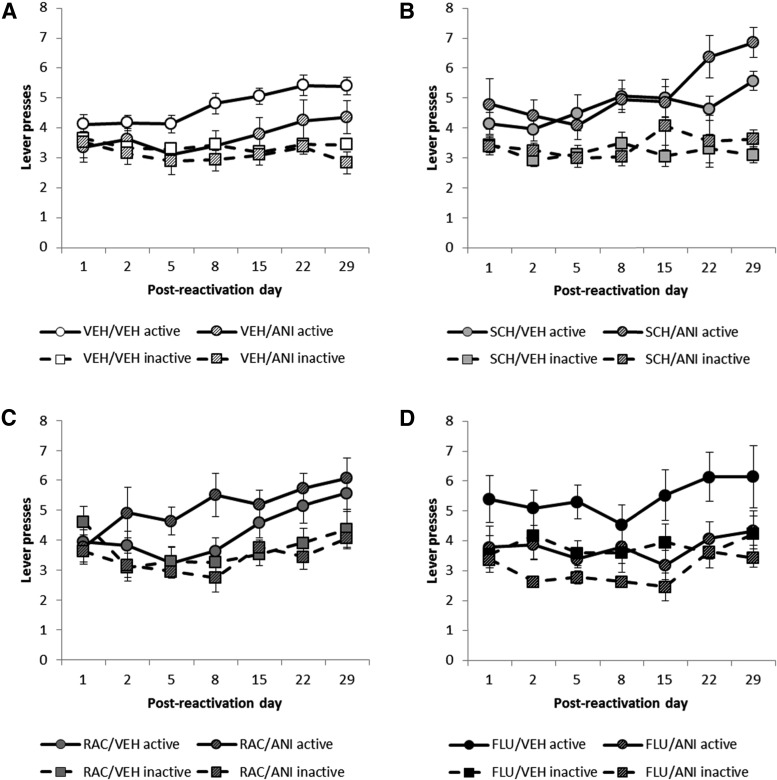
D_1_- or D_2_-subtype-selective dopamine receptor antagonism prevented the destabilization of CS−sucrose memory. ***A***, The protein synthesis inhibitor anisomycin (ANI), given immediately after CS−sucrose memory reactivation, disrupted memory reconsolidation when it followed an intra-BLA vehicle (VEH) infusion prior to reactivation, consequently preventing the CS from acting as a conditioned reinforcer during the test sessions. ***B***, ***C***, Antagonism at D_1_ dopamine receptors with SCH23390 (SCH; ***B***) or antagonism at D_2_ dopamine receptors with raclopride (RAC; ***C***) prior to reactivation prevented the amnestic effect of ANI, allowing the CS to act as a conditioned reinforcer during testing, consistent with a blockade of memory destabilization. ***D***, Nonselective antagonism at dopamine receptors with α-flupenthixol (FLU) prior to reactivation did not prevent the amnestic effect of ANI administered post-reactivation. Data are square-root transformed and shown as mean ± SEM. Group sizes are as in Figure 2, with the same animals tested in each session.

The destabilization of a previously well-consolidated CS−sucrose memory was impaired by infusion of either the D_1_- or the D_2_-dopamine receptor antagonist. However, infusion of the nonselective antagonist α-flupenthixol had no effect on memory destabilization (Fig. 3). The data were transformed prior to analysis (see Materials and Methods) and were analyzed in a single, omnibus ANOVA before planned comparisons were made between animals receiving post-reactivation infusions of VEH and ANI for each experimental condition given a different dopamine receptor antagonist prior to reactivation.

The previously sucrose-associated CS was only capable of functioning as a conditioned reinforcer, measured by its ability to support ANR, in some of the experimental groups, depending upon the treatments that were received immediately before and immediately after the memory reactivation session (Fig. 3) (Drug1 × Drug2: *F*_(3,69)_ = 3.40, *p* = 0.023, η^2^ = 0.13; Lever × Drug1 × Drug2: *F*_(3,69)_ = 3.11, *p* = 0.032, η^2^ = 0.12). Importantly, there were no simple main effects of dopamine receptor antagonism (Drug1: *F*_(3,69)_ = 1.24, *p* = 0.30; Lever × Drug1: *F*_(3,69)_ = 1.16, *p* = 0.33) or protein synthesis inhibition (Drug2: *F*_(1,69)_ = 2.67, *p* = 0.11; Lever × Drug2: *F* < 1) on the memory; rather, the capacity of the CS to act as a conditioned reinforcer depended upon both treatments, consistent with a blockade of the memory destabilization process (Ben Mamou et al., 2006; Milton et al., 2013).

As shown previously with NMDA receptor (NMDAR) antagonism at memory reactivation (Milton et al., 2008), the instrumental nosepoke response previously associated in training with delivery of the CS and sucrose reinforcer was not impaired at test by the treatments that disrupted the pavlovian CS−sucrose memory (Table 3). There were no simple effects of either dopamine receptor antagonism (Drug1: *F* < 1) or protein synthesis inhibition (Drug2: *F*_(1,69)_ = 1.44, *p* = 0.23) on nosepoking behavior during testing, and unlike responding for the CS, there was no interaction between the two treatments (Drug1 × Drug2: *F*_(3,69)_ = 1.72, *p* = 0.17). Although the omnibus ANOVA indicated that pre-reactivation treatment with a dopamine receptor antagonist altered nosepoking behavior across the course of ANR testing (Session × Drug1: *F*_(16.7,386)_ = 1.71, *p* = 0.039, η^2^ = 0.069), this was a very small effect and there were no differences between animals treated with any of the dopamine receptor antagonists in any of the individual test sessions when Šidák-corrected pairwise comparisons were conducted (all *p*’s > 0.072).

**Table 3:** Nosepokes made during testing of the acquisition of a new instrumental response for conditioned reinforcement. Data are presented as mean ± s.e.m. and are given to 3sf.

Post-reactivation day	1	2	5	8	15	22	29
VEH/VEH	54.1 ± 5.79	52.2 ± 5.60	47.5 ± 3.95	49.8 ± 3.98	56.9 ± 3.65	45.2 ± 3.99	38.3 ± 3.05
VEH/ANI	39.3 ± 6.07	42.9 ± 6.92	40.3 ± 5.21	44.9 ± 5.98	36.6 ± 4.92	38.9 ± 4.82	36.7 ± 6.26
SCH/VEH	61.1 ± 11.5	43.0 ± 4.30	39.5 ± 4.99	50.5 ± 8.82	38.5 ± 3.58	48.8 ± 13.4	37.6 ± 8.21
SCH/ANI	43.0 ± 9.49	35.3 ± 5.12	33.2 ± 4.90	33.8 ± 4.44	35.0 ± 5.65	32.3 ± 5.82	29.8 ± 6.40
RAC/VEH	33.1 ± 4.98	32.6 ± 4.57	36.5 ± 5.54	39.4 ± 4.85	50.8 ± 4.63	42.0 ± 5.04	40.5 ± 4.56
RAC/ANI	39.3 ± 6.47	44.9 ± 12.5	47.4 ± 9.36	66.4 ± 9.72	55.3 ± 5.56	56.3 ± 9.96	43.3 ± 6.75
FLU/VEH	45.7 ± 8.73	40.2 ± 5.54	50.0 ± 8.11	45.2 ± 8.27	68.0 ± 23.7	43.2 ± 5.17	54.2 ± 8.36
FLU/ANI	48.4 ± 11.0	33.6 ± 4.45	40.0 ± 6.75	33.4 ± 2.90	35.6 ± 4.92	47.3 ± 11.1	42.9 ± 7.57

### Protein synthesis inhibition with anisomycin prevented the restabilization of the CS−sucrose memory, preventing the CS from subsequently acting as a conditioned reinforcer in groups that received vehicle prior to reactivation

Consistent with previous data (Lee et al., 2005), inhibition of protein synthesis with post-reactivation anisomycin infusions into the BLA impaired the capacity of the previously sucrose-paired CS to act as a conditioned reinforcer at subsequent test (Fig. 3*A*) (ANI: *F*_(1,33)_ = 5.33, *p* = 0.027, η^2^ = 0.14). Šidák-corrected pairwise comparisons revealed that while the VEH/VEH group showed discriminated responding on the active (CS-producing) lever [*p* < 0.001] the VEH/ANI group did not discriminate (*p* = 0.18). Therefore, the pavlovian CS−sucrose memory destabilized in both groups, and was prevented from restabilizing and subsequently persisting in the VEH/ANI group.

The number of nosepoke responses made during the ANR testing sessions (Table 3) was not affected by protein synthesis inhibition immediately after memory reactivation (ANI: *F*_(1,33)_ = 2.05, *p* = 0.16; Session × ANI: *F* < 1), indicating that although the pavlovian CS−sucrose memory was rendered sensitive to disruption during the memory reactivation session, the instrumental memory of responding for sucrose was not.

### D_1_-subtype-selective dopamine receptor antagonism with SCH23390 prior to reactivation blocked the amnestic effect of anisomycin administered after reactivation

Administration of the D_1_-selective dopamine receptor antagonist SCH prior to the memory reactivation session prevented the destabilization of the CS−sucrose memory (Fig. 3*B*). A significant interaction between SCH and ANI indicates that SCH prevented the amnestic effect of post-reactivation anisomycin (SCH × ANI: *F*_(1,45)_ = 6.75, *p* = 0.013, η^2^ = 0.13; ANI: *F* < 1; Lever × ANI: *F* < 1; Lever × Session × ANI: *F*_(2.99,35.9)_ = 1.60, *p* = 0.21). In contrast to VEH/ANI experimental group, all animals that received SCH prior to memory reactivation showed discriminated responding for conditioned reinforcement on the active lever during subsequent testing, with increased active lever pressing over sessions (Lever: *F*_(1,12)_ = 36.7, *p* < 0.001, η^2^ = 0.75; Lever × Session: *F*_(2.99,35.9)_ = 3.88, *p* = 0.017, η^2^ = 0.24). This shows that the CS−sucrose memory remained intact despite the post-reactivation administration of a drug that would otherwise have disrupted memory restabilization. Similarly, protein synthesis inhibition following SCH treatment did not reduce the number of nosepokes (Table 3) made during the test sessions (ANI: *F*_(1,12)_ = 1.55, *p* = 0.24; Session × ANI: *F* < 1), indicating that the instrumental memory, like the pavlovian memory, remained intact.

### D_2_-subtype-selective dopamine receptor antagonism with raclopride prior to reactivation prevented the amnestic effect of anisomycin administered after reactivation

The D_2_-selective dopamine receptor antagonist RAC also prevented the amnestic effect of post-reactivation anisomycin, consistent with a blockade of memory destabilization (RAC × ANI: *F*_(1,46)_ = 7.26, *p* = 0.01, η^2^ = 0.14) (Fig. 3*C*). Both groups showed discriminated responding on the active (CS-producing) lever (Lever: *F*_(1,13)_ = 23.1, *p* < 0.001, η^2^ = 0.64) and interestingly, though the RAC/VEH and RAC/ANI groups responded differently (Lever × ANI: *F*_(1,13)_ = 6.04, *p* = 0.029, η^2^ = 0.32), this was due to better discrimination in the RAC/ANI group compared with the RAC/VEH group. While the RAC/ANI group discriminated between the active and inactive lever from the second testing session (all *p*’s < 0.032 from Session 2 onwards), the RAC/VEH group did not reliably discriminate between the levers until Session 5 (*p*’s < 0.042). However, ANI did not produce amnesia (ANI: *F* < 1), indicating a blockade of memory destabilization by RAC. Furthermore, there were no differences between the RAC/VEH and RAC/ANI groups in the numbers of nosepokes (Table 3) made during the test sessions (ANI: *F*_(1,13)_ = 2.64, *p* = 0.13; Session × ANI: *F* < 1), indicating that, as for D_1_-selective dopamine receptor antagonism, D_2_R antagonism left instrumental responding intact and prevented the destabilization of the pavlovian memory.

### Protein synthesis inhibition with anisomycin following reactivation still produced amnesia when the nonselective dopamine receptor antagonist α-flupenthixol was administered prior to reactivation

Administration of the mixed D_1_/D_2_ dopamine receptor antagonist FLU prior to memory reactivation, by contrast to D_1_- and D_2_-subtype-selective dopamine receptor antagonism, did not prevent the destabilization of the CS−sucrose memory (Fig. 3*D*), as post-reactivation infusion of anisomycin resulted in amnesia independent of the presence of FLU (FLU × ANI: *F*_(1,44)_ = 2.26, *p* = 0.14, η^2^ = 0.05; ANI: *F*_(1,11)_ = 7.48, *p* = 0.019, η^2^ = 0.41). While the animals in the FLU/VEH group discriminated between the active (CS-producing) and inactive lever during testing (*p* = 0.018), those in the FLU/ANI group did not (*p* = 0.12). Thus, pre-reactivation administration of FLU did not prevent the amnestic effect of ANI. Furthermore, as for VEH-treated animals, the number of nosepoke responses (Table 3) made during the testing sessions was unaffected by protein synthesis inhibition in conjunction with memory reactivation (ANI: *F*_(1,11)_ = 1.36, *p* = 0.27; Session × ANI: *F* < 1). Thus, in contrast with D_1_R-selective or D_2_R-selective antagonism, nonselective dopamine receptor antagonism affected neither destabilization nor restabilization of the pavlovian memory or the instrumental memory.

### Dopamine receptor antagonism prior to memory reactivation did not affect subsequent responding for a conditioned reinforcer itself, but altered the normally amnestic effect of anisomycin in some experimental groups

Dopamine receptor antagonism prior to reactivation did not itself alter subsequent responding for conditioned reinforcement. Comparing groups that received VEH following reactivation, there were no differences in subsequent responding for conditioned reinforcement, regardless of which dopamine receptor antagonist had been given prior to the memory reactivation session (Drug1: *F* < 1; Lever × Drug1: *F*_(3,43)_ = 1.43, *p* = 0.25; Session × Drug1: *F*_(15.6,224)_ = 1.11, *p* = 0.34; Lever × Session × Drug1: *F* < 1).

In contrast, dopamine receptor antagonism prior to memory reactivation did affect the capacity of the protein synthesis inhibitor anisomycin to induce amnesia following reactivation. Comparing groups that received ANI following reactivation, there was a difference in subsequent responding for conditioned reinforcement, depending on which dopamine receptor antagonist had been administered prior to reactivation. This was reflected by differences in overall lever pressing during the ANR sessions (Drug1: *F*_(3,26)_ = 3.01, *p* = 0.049, η^2^ = 0.26) and a trend towards differences in pressing the active and inactive levers (Lever × Drug1: *F*_(3,26)_ = 2.53, *p* = 0.079, η^2^ = 0.23). Follow-up ANOVAs comparing lever pressing during ANR for individual experimental groups revealed that although the VEH/ANI-treated (Lever: *F*_(1,8)_ = 2.13, *p* = 0.18) and FLU/ANI-treated (Lever: *F*_(1,7)_ = 3.06, *p* = 0.12) did not discriminate between the active and inactive levers during ANR testing, the SCH/ANI-treated (Lever: *F*_(1,5)_ = 29.6, *p* = 0.003, η^2^ = 0.86) and RAC/ANI-treated (Lever: *F*_(1,6)_ = 39.6, *p* = 0.001, η^2^ = 0.87) animals pressed the active lever, which was reinforced by presentation of the previously sucrose-associated CS, more during the test sessions.

## Discussion

The data presented here demonstrate that blocking dopaminergic signaling within the BLA either at the D_1_R with SCH23390 or at the D_2_R with raclopride prevented the amnesia that normally follows post-reactivation protein synthesis inhibition. Therefore, the CS−sucrose memory, retrieved and behaviorally expressed during the memory reactivation session, did not require protein synthesis in order to restabilize and subsequently persist—consistent with the hypothesis that activity at either D_1_Rs or D_2_Rs is required for destabilization of the CS−sucrose memory. That the memory’s persistence was independent of protein synthesis cannot be attributed to the parameters of the memory reactivation session, since rats having received intra-amygdala vehicle infusions prior to reactivation were subsequently amnesic at test following post-reactivation administration of the protein synthesis inhibitor anisomycin. Furthermore, nonselective antagonism at dopamine receptors with α-flupenthixol did not block the subsequent amnestic effect of post-reactivation anisomycin. Although it is possible that a higher dose of α-flupenthixol may have prevented anisomycin-induced amnesia, the dose of α-flupenthixol used in these experiments is sufficiently high to be behaviorally effective, but not so high so as to produce locomotor side effects (Di Ciano and Everitt, 2004). Thus, these findings may suggest that memory destabilization requires a differential state of activation of D_1_Rs and D_2_Rs in the BLA in order to proceed.

We speculate that the requirement for dopamine in the destabilization of the CS−sucrose memory is linked to its role in signaling prediction error. It should be noted, however, that in addition to its hypothesized role in prediction error, dopaminergic signaling has also been theoretically implicated in the encoding of hedonic processes (Wise and Rompre, 1989) and the attribution of incentive salience (Berridge and Robinson, 1998). We suggest that it is unlikely that the data presented here can be accounted for in terms of these other hypothesized functions of dopamine. Firstly, it is unlikely that the administration of dopamine receptor antagonists impaired motivation for the sucrose reward, thereby devaluing the conditioned reinforcer associated with it. The evidence for dopamine in encoding hedonic processing is mixed at best (Salamone and Correa, 2012), and in these experiments, the dopamine receptor antagonists were administered only once, during a memory reactivation session in which the primary reinforcer was unavailable, making it unlikely that dopamine receptor antagonism could have led to devaluation effects. Secondly, because responding for the CS during the memory reactivation session was unaffected by the prior administration of the dopamine receptor antagonists (Fig. 2), it is unlikely that these drugs produced acute effects on the salience of the CS, or on the motivation to work for the CS, that would only have become apparent in the subsequent ANR testing phase beginning 24 h later. Thus, we suggest that the data presented here are most consistent with a role for dopamine in signaling prediction error during the destabilization of an appetitive memory.

Although prediction error has previously been linked theoretically to reconsolidation (Pedreira et al., 2004; Eisenhardt and Menzel, 2007; Forcato et al., 2007; Sevenster et al., 2013), and it has also been suggested that dysregulation of dopaminergic signaling underlying prediction error may lead to aberrant reconsolidation in both schizophrenia (Corlett et al., 2009) and drug addiction (Tronson and Taylor, 2013), this is the first empirical demonstration that signaling at dopamine receptors, specifically within the BLA, prevents the destabilization of a reconsolidating memory, as measured by a lack of effect of post-reactivation anisomycin. Although dopaminergic projections from the ventral tegmental area have recently been shown to be necessary for memory destabilization (Reichelt et al., 2013), the current study demonstrates in a temporally and spatially more selective manner that activity at dopamine receptors is required within the BLA, specifically at the time when the memory would normally destabilize at reactivation. However, the present data are also challenging in two respects: firstly, the finding that either D_1_R antagonism or D_2_R antagonism prevented memory destabilization appears counterintuitive, as D_1_Rs and D_2_Rs have generally been seen to produce opposing effects on intracellular signaling pathways, at least in the striatum (Stoof and Kebabian, 1981); and secondly, the finding that nonselective dopamine receptor antagonism did not prevent destabilization, when either of the selective receptor antagonists did, requires explanation.

The similar effect of intra-amygdala D_1_R and D_2_R antagonism has been seen previously. Electrophysiological studies of BLA neurons have shown that both D_1_R and D_2_R agonists produce an overall reduction in BLA output (Rosenkranz and Grace, 1999), and that antagonism at either subtype of dopamine receptor reduces the overall excitability of BLA projection neurons (Kröner et al., 2005). Furthermore, behaviorally, both D_1_R antagonists (Lamont and Kokkinidis, 1998; Guarraci et al., 1999; Greba and Kokkinidis, 2000) and D_2_R antagonists (Guarraci et al., 2000) have anxiolytic effects, suggesting that their overall effect on amygdala function is similar. One model to account for these similar effects, proposed by Ehrlich and colleagues (2009), is that despite D_1_Rs being expressed on projection neurons (Pickel et al., 2006), they are also expressed on the lateral intercalated cells; since D_2_Rs are mainly expressed on local fast-spiking inhibitory interneurons, both dopamine receptor subtypes are expressed on cells that inhibit amygdala projection neurons. Stimulation of either of these dopamine receptor subtypes leads to a reduction in interneuron activity, and therefore disinhibition of BLA projection neurons (Bissière et al., 2003; Marowsky et al., 2005); thus, antagonism at D_1_Rs or D_2_Rs would be predicted to increase interneuron activity, reduce the activity of the BLA projection neuron, and so impair synaptic plasticity processes—we would argue including memory destabilization—within the BLA. Furthermore, the effect of D_1_R antagonism may be twofold; directly reducing excitation of pyramidal neurons (Kröner et al., 2005) and indirectly increasing pyramidal cell inhibition through interneurons (Ehrlich et al., 2009). The action of dopamine on these hypothesized networks within the amygdala is consistent with our findings, reported here, that either D_1_R or D_2_R antagonism was sufficient to prevent the destabilization of the reconsolidating memory.

The model proposed by Ehrlich et al. (2009) may also provide an explanation for the lack of effect of α-flupenthixol. In contrast to the disinhibitory effects of D_1_R-selective (Kröner et al., 2005; Marowsky et al., 2005) or D_2_R-selective (Bissière et al., 2003) agonism, it has been demonstrated previously that simultaneous agonism at both D_1_Rs and D_2_Rs results in an increase in spontaneous inhibition within the BLA inhibitory network (Lorétan et al., 2004). In the same manner, antagonizing both subtypes of dopamine receptor with α-flupenthixol may, somewhat paradoxically, disinhibit the network without interfering with destabilization.

The role of D_1_Rs and D_2_Rs in modulating amygdala activity, and elucidation of the network dynamics, require further study. However, the results of the experiments reported here clearly demonstrate that dopaminergic signaling is a requirement for reactivation-induced memory destabilization. Destabilization and prediction error therefore have a shared dependence upon dopaminergic signaling, as a retrieved memory requires prediction error in order to destabilize (Sevenster et al., 2013), although we did not directly test the requirement for prediction error in destabilization in these experiments. Whether dopaminergic signaling is sufficient to engage destabilization in the absence of prediction error, and how dopaminergic mechanisms link to already established destabilization mechanisms such as protein degradation (Kaang et al., 2009) and activation of the GluN2B-subtype of NMDAR (Ben Mamou et al., 2006; Milton et al., 2013) are unresolved questions, although it has been suggested that dopamine receptors can influence GluN2B-NMDAR phosphorylation (Yang et al., 2012). Furthermore, enhancing the destabilization process may increase the lability of maladaptive emotional memories, thereby facilitating the treatment of psychiatric disorders such as addiction, where retrieved CS−drug memories can elicit drug craving and relapse.

## Synthesis

The decision was a result of the Reviewing Editor Carmen Sandi and the peer reviewers coming together and discussing their recommendations until a consensus was reached. A fact-based synthesis statement explaining their decision and outlining what is needed to prepare a revision is listed below. The following reviewers agreed to reveal their identity: Paul E. M. Phillips and Devin Mueller

Although there is a large literature examining the restabilization of memory retraces after retrieval, very few studies have examined the initial destabilization. Thus, this study provides important insight into some of the mechanisms of destabilization of memory following retrieval. Specifically, it provides new information on dopamine and reconsolidation. Using psychopharmacological approaches in rats, the study shows that antagonizing dopamine receptors (either D1 or D2) in the basolateral amygdala specifically, prevents the destabilization of a reconsolidating memory, as measured by a lack of effect of the administration of the protein synthesis inhibitor, anisomycin, given shortly after memory reactivation. Although this study does not test the requirement for prediction error in destabilization in these experiments, the authors put together a literature background and rationale that brings them to speculate about the link between these observations and the prediction error literature. However, further work will be needed in the future to precisely test whether prediction errors (and which type of prediction errors) are indeed involved in memory destabilization.

One request to the authors is to exchange 'pavlovian' for 'Pavlovian' throughout the text.
